# The role of post‐translational modifications in cardiac hypertrophy

**DOI:** 10.1111/jcmm.14330

**Published:** 2019-04-04

**Authors:** Kaowen Yan, Kun Wang, Peifeng Li

**Affiliations:** ^1^ Institute for Translational Medicine, College of Medicine Qingdao University Qingdao China

**Keywords:** cardiac hypertrophy, heart failure, post‐translational modifications (PTMs)

## Abstract

Pathological cardiac hypertrophy involves excessive protein synthesis, increased cardiac myocyte size and ultimately the development of heart failure. Thus, pathological cardiac hypertrophy is a major risk factor for many cardiovascular diseases and death in humans. Extensive research in the last decade has revealed that post‐translational modifications (PTMs), including phosphorylation, ubiquitination, SUMOylation, O‐GlcNAcylation, methylation and acetylation, play important roles in pathological cardiac hypertrophy pathways. These PTMs potently mediate myocardial hypertrophy responses via the interaction, stability, degradation, cellular translocation and activation of receptors, adaptors and signal transduction events. These changes occur in response to pathological hypertrophy stimuli. In this review, we summarize the roles of PTMs in regulating the development of pathological cardiac hypertrophy. Furthermore, PTMs are discussed as potential targets for treating or preventing cardiac hypertrophy.

## 1 INTRODUCTION

The heart undergoes adaptive changes in response to long‐term overload, namely myocardial hypertrophy. Physiological hypertrophy usually happens to pregnant women or athletes. 1 However, pathological cardiac hypertrophy is usually induced by stress stimulation or disease and is a typical pathological stage of diseases such as cardiomyopathy, myocardial infarction and diabetes.^2^ Therefore, pathological cardiac hypertrophy is a predictor of many cardiovascular diseases and death in humans.

At the cellular level, the typical characteristics of pathological cardiac hypertrophy are increased cardiac muscle cell size, segregation of sarcomere structures, enhanced protein synthesis and foetal gene re‐expression.^3^ Cardiac hypertrophy is an adaptive response mediated by regulation at multiple levels, including the transcription, processing and translation of mRNAs and post‐translational modifications (PTMs).^4^ PTMs are more flexible and economical than regulation at the transcriptional level. PTMs usually regulate the activation/inactivation or degradation of pre‐existing transcripts and proteins covalently modified by enzymes, resulting in rapid changes in the functions of pre‐existing proteins, multiprotein complexes and subcellular structures in response to various physical and chemical stimuli.^5^ Owing to PTMs, cardiomyocyte does not trigger de novo synthesis of proteins at the transcriptional level, which provides an approach to the saving of energy and material resources and compensates for the temporal‐and‐spatial weaknesses caused by transcriptional regulation. In addition, PTMs act as key regulators of proteins, contributing to changes in their diversity, localization, structure, interaction, roles etc, thus providing substantial complexity and elaborate regulation to the control of cardiac hypertrophy. In the last decade, PTMs, including phosphorylation, polyubiquitination, SUMOylation, O‐GlcNAcylation methylation and acetylation, were reported to play essential roles in myocardial hypertrophy pathways. These signalling pathways include Ca^2+^/calmodulin, mitogen‐activated protein kinase (MAPK), JAK‐STAT, protein kinase C, phosphatidylinositol 3‐kinase (PI3K)/Akt, inflammation, nuclear factor‐κB (NF‐κB) and adenosine‐activated protein kinase (AMPK). Therefore, comprehensive knowledge of PTMs involved in the development of myocardial hypertrophy will provide a better understanding of the molecular regulatory mechanism of pathological hypertrophy. This, in turn, will greatly benefit rational drug utilization and provide new treatment strategies for heart failure.

## 2 PHOSPHORYLATION

MAPKs, consisting of extracellular signal‐regulated kinases (ERKs), c‐Jun N‐terminal kinases (JNKs) and p38 MAPKs, are well known to play important roles in mediating overload or pathological insult‐induced cardiac hypertrophy.^6^ For example, cardiomyocyte‐specific expression of MEK‐1 significantly induced ventricular concentric hypertrophy by phosphorylating ERK1/2 in the heart (Table 1).^7^ ERK5 was also shown to play an essential role in the development of cardiac hypertrophy.^8^ The Ca^2+^/calmodulin signalling pathway reportedly plays an important role in the occurrence of ventricular arrhythmias in hypertrophic cardiomyopathy and cardiac hypertrophy.^9^ Ersilia et al showed that the CaMKII‐ERK pathway was essential for developing cardiac hypertrophy and the impairment of their interaction provided a promising therapeutic modality to attenuate myocardial hypertrophy.^10^ Recently, activation of ERK/glycogen synthase kinase‐3(GSK3) induced by angiotensin II was shown to phosphorylate heat shock factor 1 (HSF1), resulting in degradation of RNF126, which promoted the expression of insulin‐like growth factor II receptor (IGF‐IIR) and ultimately induced myocardial hypertrophy (Table 1).^11^ Thus, targeting HSF1 could be a promising strategy to prevent pathological cardiac hypertrophy.

**Table 1:** Roles of phosphorylation in myocardial hypertrophy

Name	Target	Result	Role in heart	Reference
MEK1	ERK1/2	Activation	Induced pathological cardiac hypertrophy	7
MEK5	ERK5	Activation	Exacerbated pathological cardiac hypertrophy	8
CaMKII	ERK1/2	Activation	Induced pathological cardiac hypertrophy	10
ERK/GSK3	HSF1	Inactivation	Exacerbated pathological cardiac hypertrophy	11
RGS6	ASK1	Activation	Exacerbated pathological cardiac hypertrophy	13
PI3K	AKT	Activation	Induced pathological cardiac hypertrophy	16
MEK3 and MEK6	p38	Activation	Contributed to cardiac hypertrophy	13
MEK4 and MEK7	JNK	Activation	Contributed to cardiac hypertrophy	14
FAK	AKT	Activation	Contributed to cardiac hypertrophy	17
LKB1	AMPK	Activation	Inhibited cardiomyocyte hypertrophy	21, 23

Kojonazarov et al showed that inhibition of p38 MAPK activity improved heart function in response to pressure‐loaded right ventricular hypertrophy by suppressing transcriptional pathways, including serum response factor and myocardin‐related transcription factor A.^12^ Regulator of G protein signalling 6 (RGS6) was reported to promote cardiac hypertrophy by activating apoptosis signal‐regulating kinase1/p38 MAPK/JNK1/2 signalling.^13^ A deficiency of JNK‐interacting protein 3 could alleviate cardiac hypertrophy through inactivating the JNK pathway and might become a promising therapeutic target for treating cardiac hypertrophy and heart failure.^14^

AKT, a serine/threonine kinase, is activated and phosphorylated by PDK1 and PDK2 at residues Thr308 and Ser473 respectively.^15^ As a key molecule for cardiac hypertrophy, AKT activation can further phosphorylate many downstream proteins and thereby positively and negatively regulate diverse signalling pathways. AKT has been shown to promote cardiac hypertrophy through regulating several signalling pathways, such as PI3K/AKT/GSK3β, PI3K/AKT/mTOR and the FAK/AKT signalling.^16^, ^17^ Knockdown of protein kinase D (PKD) was shown to attenuate pressure overload‐induced cardiac hypertrophy by promoting autophagy via AKT/mTOR pathway.^19^ Dimethyl fumarate, a methyl ester of fumaric acid, is approved by the Food and Drug Administration for the treatment of relapsing/remitting multiple sclerosis and psoriasis. Dimethyl fumarate was shown to protect against ISO‐induced cardiac hypertrophy by decreasing the levels of p‐ERK1/2 and increasing the level of p‐AKT.^20^

AMPK, a serine/threonine kinase, is activated and phosphorylated by LKB1 at residue Thr172 (Table 1).^21^ AMPK activation can further phosphorylate numerous downstream proteins and thereby positively and negatively regulate diverse signalling pathways. AMPK has been shown to protect against cardiac hypertrophy by inhibiting protein synthesis and its development through a lot of downstream proteins, such as eukaryotic elongation factor‐2 (eEF2), AKT/mTOR/p70S6 kinase, SIRT1/eNOS/p53 and transcriptional regulation factors including NFAT, PPAR‐α and FOXO.^22^, ^23^ Notably, a lot of pharmacological AMPK activators, such as metformin, berberine, AICAR and A‐769662, have been shown to protect against cardiovascular diseases.^26^, ^27^ For example, berberine was reported to activate AMPK in diabetic rats, resulting in improvement of cardiac dysfunction and attenuation of cardiomyocyte hypertrophy.^28^

NF‐κB, a key transcription factor, plays an important role in diverse cellular processes, including cell survival, apoptosis, growth and differentiation.^29^ NF‐κB is tightly regulated by the inhibitory protein IκB, which is phosphorylated by the upstream IKK kinase to degrade IκB and release NF‐κB. Subsequently, activated NF‐κB is translocated to the nucleus where it activates gene expression.^30^ NF‐κB activation was shown to promote cardiac hypertrophy via activating foetal gene re‐expression. RasGAPSH3 domain‐binding proteins (G3BPs) were shown to promote isoproterenol‐induced cardiac hypertrophy via the activation of NF‐κB signalling pathway.^31^ Recently, auranofin, a 19S proteasome‐associated deubiquitinase inhibitor, was shown to attenuate cardiac hypertrophy by blocking NF‐κB activation.^32^

Many protein kinase‐mediated autophagies were shown to be involved in the development of cardiac hypertrophy. The protein kinase AKT, ERK1/2, PKA, MAPK and AMPK are related to autophagy. For instance, in the presence of growth factors, AKT inhibits initiation of autophagy by phosphorylating numerous downstream proteins, including TSC2, PRAS40, GSK3, FOXO and Beclin‐1.^33^ In contrast, AMPK is shown to induce autophagy by inhibiting mTOR signalling.^34^ However, the role of autophagy in pressure stress‐induced cardiac hypertrophy remains controversial.^1^ Xu et al showed that autophagy activated by mTOR signalling protects against cardiac hypertrophy.^35^ But Zhu et al indicated that excessive autophagy accentuates pressure stress‐induced cardiac hypertrophic responses.^36^ Therefore, the exact regulatory network followed by protein kinase‐mediated autophagy in cardiac hypertrophy needs further investigation in the future.

Together, the above‐mentioned findings suggest that phosphorylation is essential for promoting or attenuating cardiac hypertrophy in various signal pathways.

## 3 dual‐specificity MAPK phosphatases

A previous study has shown that DUSPs act as critical regulators of cardiac growth and remodelling by dynamically regulating the MAPK signalling pathway (Table 2).^37^ DUSP12 ameliorates cardiac hypertrophy via inhibiting JNK1/2 activity.^38^ DUSP8 is involved in cardiac ventricular remodelling by activating ERK1/2 signalling. Cardiac‐specific overexpression of DUSP8 causes spontaneous eccentric remodelling and ventricular dilation with heart failure.^39^ DUSP14 prevents cardiac hypertrophy and dysfunction induced by aortic banding by inactivating the TAK1/p38MAPK/JNK1/2 signalling pathway.^40^ In addition, heat shock protein 90 regulates cardiac ventricular hypertrophy through the activation of MAPK pathway.^41^ In brief, phosphorylation modifications play important roles in the regulation of cardiac hypertrophy and may prove to be promising targets for therapeutic development.

**Table 2:** Role of DUSPs in myocardial hypertrophy

DUSP	Target	Role in heart	Reference
DUSP1	ERK1/2,JNK1/2,p38	Attenuated cardiac hypertrophy	42
DUSP4	ERK1/2	Positively regulated cardiac hypertrophy	43
DUSP8	ERK1/2,JNK1/2,p38	Positively regulated cardiac hypertrophy	39
DUSP12	JNK1/2	Attenuated cardiac hypertrophy	38
DUSP14	JNK1/2,p38	Attenuated cardiac hypertrophy	40

## 4 UBIQUITINATION

**Figure 1:**
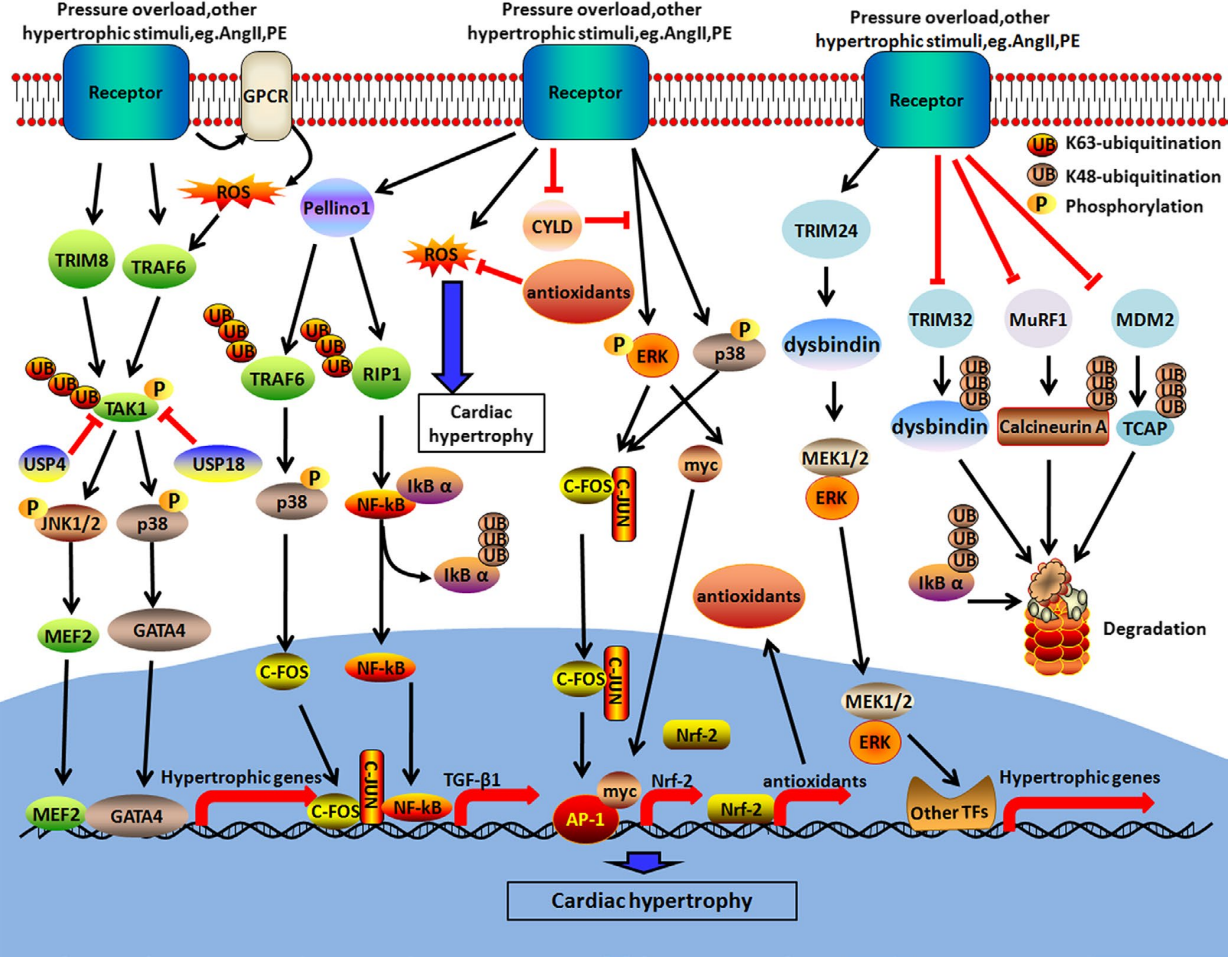
Ubiquitination‐mediated signalling pathways of cardiac hypertrophy. Ubiquitination plays an important role in cardiac hypertrophy by regulating the TAK1‐JNK1/2/p38, NF‐κB signalling, Ca^2+^/calmodulin, oxidation stress, ERK signalling pathways. In these pathways, pressure overload or other hypertrophic stimuli can induce E3 ligases or DUBs to activate MAPKs or other signalling pathways, ultimately regulating nuclear transcription factors to promote growth

Ubiquitination, a widely distributed PTM of proteins, regulates the timely functions of proteins. Recently, ubiquitin‐proteasome system (UPS) proteins, E3 ligases and deubiquitylation enzymes (DUBs) were found to play important roles in the development of cardiac hypertrophy (Figure 1; Table 3).^42^ Studies found that K63‐linked polyubiquitination of TAK1 triggered by the E3 ligase, TRIM8, leads to pathological hypertrophy. Thus, suppression of cardiac TRIM8 expression could attenuate the induction of cardiac hypertrophy.^43^, ^44^ Li et al reported that the level of TRAF6 in hypertrophic human and mouse hearts was increased. Furthermore, heart‐specific overexpression of TRAF6 aggravated myocardial hypertrophy in response to pressure overload or stimulation with angiotensin II. In terms of the mechanism, auto‐ubiquitination of TRAF6 triggered by reactive oxygen species promoted TAK1 ubiquitination, which induced cardiac hypertrophy.^45^ Recent studies reported that DUB was involved in regulating the development of cardiac hypertrophy through the TAK1 signalling pathway.^46^ For example, ubiquitin‐specific protease 4 (USP4) inhibited pathological cardiac hypertrophy and dysfunction by hydrolysing the K63 ubiquitination of TAK1, resulting in the suppression of TAK1‐JNK1/2/p38 signalling.^47^ In addition, Ying et al reported that ubiquitin‐specific protease 18 (USP18) attenuated cardiac hypertrophy by specifically removing the K63‐linked polyubiquitination of TAK1, bringing about inactivation of TAK1‐p38/JNK1/2 signalling pathway.^48^ USP14, a DUB of the 19S proteasome subunit, was shown to promote cardiac hypertrophic responses through enhancing GSK‐3β phosphorylation, suggesting that USP14 may be a potential therapeutic target to treat cardiac hypertrophy.^49^ Previous studies have shown that UPS plays an important role in quality control mechanisms of protein production and UPS insufficiency may lead to heart failure.^50^, ^51^ Notably, whether UPS regulates heart failure by activating or inhibiting the autophagy pathway remains controversial.^52^ Recently, proteasome inhibitors, MG132 and bortezomib, were shown to attenuate cardiac hypertrophy induced by cholesterol through inhibiting the activation of ERK and Akt signalling.^53^ Rapamycin, an inhibitor of mTOR, was shown to protect against cardiac hypertrophy by promoting myocardial autophagy through the MEK/ERK/Beclin‐1 pathway.^54^

**Table 3:** Roles of E3 ligases and DUBs in myocardial hypertrophy

Name	Target	Result	Mechanism	Role in heart	Reference
TRIM8	TAK1	Activation	K63‐linked polyubiquitination of TAK1, activation of NF‐κB, (TAK1)‐p38/JNK signalling pathways	Contributed to pathological cardiac hypertrophy	45, 46
TRAF6	TAK1	Activation	K63‐linked polyubiquitination of TAK1 and activation of TAK1 signalling pathways	Exacerbated pathological cardiac hypertrophy	47
USP4	TAK1	Inactivation	Deubiquitination of TAK1 and suppression of (TAK1)‐(JNK1/2)/P38 signalling	Negatively regulated pathological cardiac hypertrophy	49
USP18	TAK1	Inactivation	Deubiquitination of TAK1and inhibition of TAK1‐p38/JNK1/2 activation	Inhibited cardiomyocyte hypertrophy	50
Pellino1	RIP1 and TRAF1	Activation	K63‐linked polyubiquitination of RIP1 and TRAF1, activation of NF‐κB, p38 and AP‐1 signalling pathways	Contributed to cardiac fibroblast activation	57
CYLD	unknown	unknown	Inactivation of ERK and p38/AP1 and suppressed Nrf2 expression	Inhibited cardiac maladaptive remodelling and dysfunction	58
USP14	unknown	Activation	Phosphorylated GSK‐3β	Contributed to cardiac hypertrophy	51
MuRF1	TRa	Inactivation	Mono‐ubiquitinated TRα and inhibited TRα activity	Inhibited T3‐induced cardiac hypertrophy	59
MuRF1	Calcineurin A	Degradation	K48‐linked polyubiquitination and degradation of Calcineurin A	Negatively regulated pathological cardiac hypertrophy	60
MDM2	TCAP	Degradation	K48‐linked polyubiquitination and degradation of TCAP	Attenuated cardiac hypertrophy	61, 62
TRIM24	Unknown	Activation	Stabilized dysbindin	Contributed to pathological cardiac hypertrophy	65
TRIM32	dysbindin	Degradation	Degraded dysbindin	Prevented pathological cardiac hypertrophy	64

Cardiac fibrosis‐induced pressure overload is an important step of maladaptive hypertrophy and ubiquitination of TRAF6 and RIP1, mediated by ligase E3 Pellino1, contributes to the activation of NF‐κB and AP‐1, resulting in increased expression of transforming growth factor‐β1 in cardiac fibroblasts (Figure 1).^55^ In addition, pressure overload‐induced cardiac maladaptive remodelling and dysfunction were mediated by deubiquitinating enzyme CYLD, which contributes to interrupt the ERK‐ and p38‐/AP‐1 and c‐Myc pathways, resulting in suppressing expression of Nrf2 and Nrf2‐operated antioxidative capacity.^56^ Furthermore, deubiquitinating enzyme USP14 suppressed the progression of cardiac hypertrophy by increasing phosphorylation of glycogen synthase kinase‐3β.^49^

Recently, the E3 ubiquitin ligase, Muscle‐specific RING finger protein‐1 (MuRF1), was reported to mono‐ubiquitinate thyroid hormone receptor α (TRα) to enhance its interaction with CAP350 and transcriptional activity in the nuclear compartment.^57^ MuRF1 was also reported to attenuate pathological cardiac hypertrophy via promoting degradation of calcineurin A.^58^ In addition, TCAP, which is down‐regulated by the E3 ubiquitin ligase, MDM2, is involved in cardiac hypertrophy (Figure 1).^59^ Moreover, Hauck et al observed that cardiac‐specific knockout of MDM2 resulted in spontaneous cardiac hypertrophy and early death in mice through the generation of reactive oxygen species (ROS).^60^ Consistent with this, cardiomyocyte hypertrophy induced by therapy with the alpha‐agonist, phenylephrine or endothelin‐1, was attenuated by overexpression of MDM2.^61^ Therefore, MDM2 may be a promising and effective target for treating heart failure. Likewise, E3 ligase tripartite motif 32 (TRIM32) has a protective role in aortic banding‐induced pathological cardiac hypertrophy by interrupting Akt signalling pathways.^62^ TRIM32 attenuates cardiomyocyte hypertrophy by regulating dysbindin protein levels, whereas the effect of TRIM24 is the opposite (Table 3).^63^

Overall, these findings show that ubiquitination modifications play an essential role in the development of cardiac hypertrophy progression and have important implications for the development of antihypertrophy drugs targeting E3 ligases and DUBs.

## 5 SUMOylation

**Figure 2:**
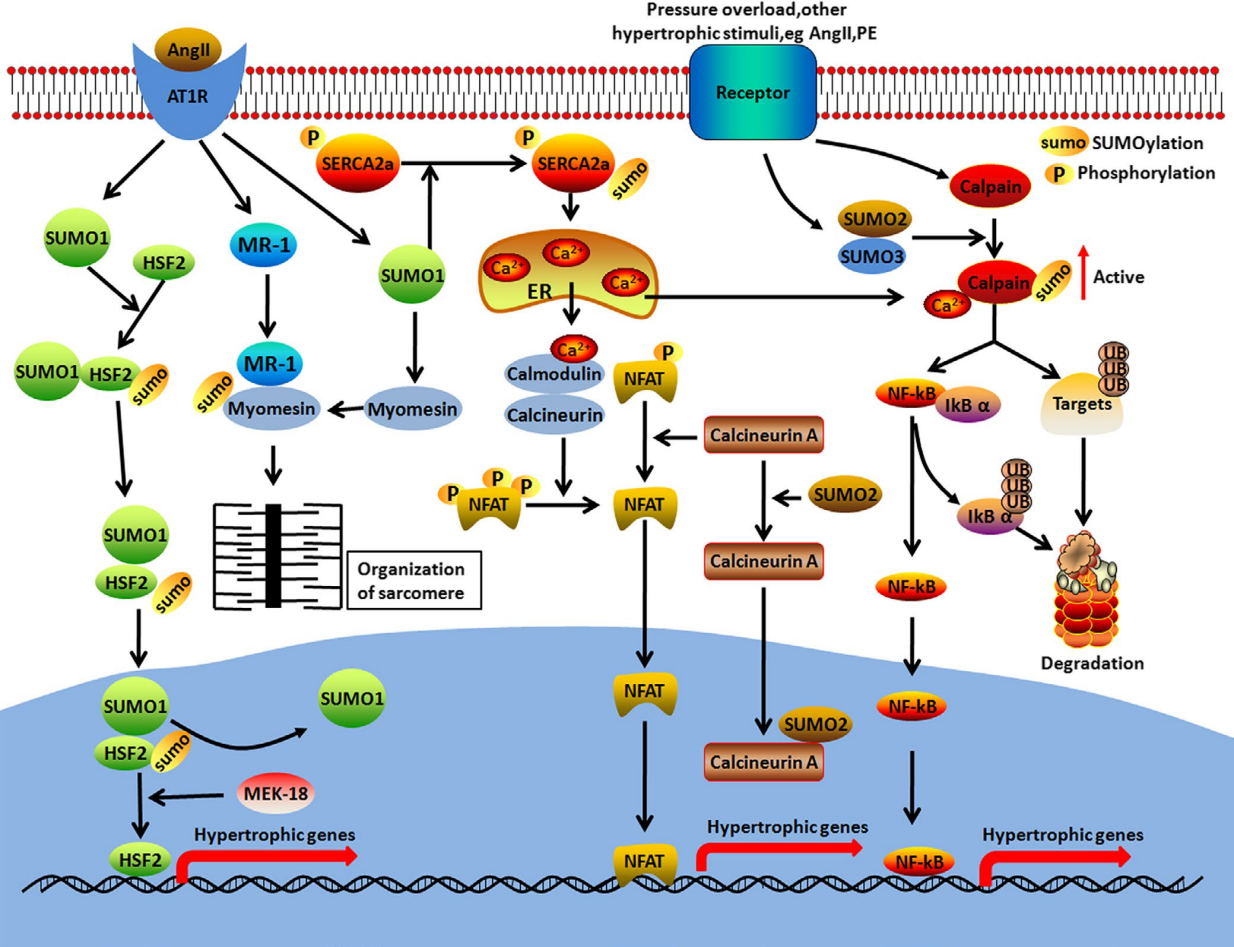
SUMOylation‐mediated signalling pathway of cardiac hypertrophy. SUMOylation plays an important role in cardiac hypertrophy by regulating the Ca^2+^/calmodulin, NF‐κB and other signalling pathways

The small ubiquitin‐like modifier (SUMO) system catalyses classical ubiquitin‐like post‐translational protein modifications that are universally involved in cellular activities such as cell cycle regulation, genome stabilization, chromatin remodelling and transcription.^64^ SUMOylation is also involved in cardiovascular diseases including cardiac hypertrophy.^65^ For example, SUMO‐1 is involved in heart failure by specifically mediating SUMOylation of SERCA2a (Table 4). Interestingly, SUMO‐1 is significantly reduced in mice and human patients with heart failure and heart failure was observed in mice following the deletion of cardiomyocyte‐specific SUMO‐1. As a result, the SUMOylation of cardiac SERCA2a was significantly decreased (Figure 2). Studies have also shown that cardiomyocyte‐specific overexpression of SUMO‐1 with AAV9 reduced the cardiac hypertrophy phenotype.^65^, ^66^ Targeting SERCA2a with adeno‐associated vector type 1 encoding SERCA2a (AAV1.SERCA2a) is considered as a new therapeutic target to treat heart failure.^67^ The initial Phase II of the Calcium Upregulation by Percutaneous Administration of Gene Therapy in Cardiac Disease (CUPID) trials delivering the SERCA2a gene for treatment of heart failure has shown potential clinical benefits. Although subsequent CUPID‐2 studies did not meet the primary or any secondary endpoints, overexpression of SERCA2a via gene transfer continues to be a promising therapeutic strategy for the treatment of heart failure.^68^ It is to be noted that SUMOylation of SERCA2a was shown to be reduced along with low SUMO1 expression in the failing heart. Thus, SUMOylation of SERCA2a activated via small molecules or enforced expression of SUMO1 with gene transfer may be a new therapeutic approach to treat heart failure.^69^ The SUMOylation of HSF2 mediated by SUMO‐1 was reported to attenuate myocardial hypertrophy. The expression of MEL‐18 is up‐regulated in response to treatment with angiotensin II, resulting in the deSUMOylation of HSF2 by the removal of SUMO‐1. This increases the expression of IGF‐IIR and induces hypertrophy (Table 4).^70^ In addition, Wang et al reported that the overexpression of myofibrillogenesis regulator 1(MR‐1) directly induced myocardial hypertrophy by enhancing the SUMOylation of myomesin‐1 (Figure 2).^71^

**Table 4:** Roles of SUMOylation in myocardial hypertrophy

Name	Target	Result	Role in heart	Reference
SUMO1	SERCA2a	Activation	Induced pathological cardiac hypertrophy	65, 66
SUMO1	HSF2	Activation	Induced pathological cardiac hypertrophy	70
SUMO1	Myomesin	Activation	Exacerbated pathological cardiac hypertrophy	71
SUMO2/3	Calpain	Activation	Exacerbated pathological cardiac hypertrophy	72, 73

In contrast to these findings, the activation of calcineurin/nuclear factor of activated T cell (NFAT) signalling, and cardiomyocyte hypertrophy induced by SUMO2, are independent of SUMOylation. SUMO2 tethers calcineurin activation to the nucleus in cardiomyocyte, facilitating the activation of NFAT to induce higher expression levels of hypertrophy‐related genes and significantly increase the cell surface area (Figure 2).^72^ Recently, Kim et al demonstrated that the SUMO2‐3 conjugation promoted the degradation of calpain‐calpastatin in failing human hearts (Table 4).^73^ Calpain mediates myocardial hypertrophy and remodelling mainly through two signalling pathways: hydrolysing calcineurin (CaN) to generate its active fragments or hydrolysing the CaN endogenous inhibitor, Cain/Cabinl, to activate the CaN signalling pathway and cleavage of IκBα to activate myocardial NF‐κB (Figure 2).^74^, ^75^ UBC9 and SUMO E2 ligase play important roles in enhancing the expression of several proteins that reside in the endoplasmic reticulum.^76^ Furthermore, the cardiomyocyte‐specific expression of UBC9 significantly improves cardiac function by increasing SUMOylation and autophagic flux in transgenic mice.^77^ In general, SUMOylation is essential for cardiac function and E3 SUMO‐protein ligases and SUMO conjugating enzymes are potential antihypertrophy drug targets.

## 6 O‐GlcNAcylation

O‐GlcNAcylation is the O‐linked attachment of the monosaccharide, β‐linked *N*‐acetyl‐glucosamine (O‐GlcNAc), to cytoplasmic, nuclear and mitochondrial proteins. It is a PTM that regulates cardiovascular disease.^78^ O‐GlcNAcylation induced by high glucose is essential for the progression of cardiac hypertrophy via increased expression of ERK1/2 and cyclin D2.^79^ The activation of AMPK pathway inhibits cardiac hypertrophy by reducing O‐GlcNAcylation in vivo.^80^ Global cardiac protein O‐GlcNAc signalling is increased in various aetiologies of cardiac hypertrophy and failure.^81^

Olson et al showed that overexpression of c‐Myc promoted cardiac hypertrophy and increased O‐GlcNAc levels.^82^ While c‐Myc knockout repressed pressure overload‐induced cardiac hypertrophy and decreased O‐GlcNAc levels. O‐GlcNAcylation stabilized c‐Myc and thus increased its transcriptional activity, consequently activating the foetal gene program to induce cardiac hypertrophy.^83^ Sp1, a transcription factor involved in the development of myocardial hypertrophy, has multiple O‐GlcNAcylation sites.^84^ It has also been shown that insulin‐induced O‐GlcNAcylation of Sp1 triggers its nuclear translocation where it is partially or wholly deglycosylated, then phosphorylated to activate foetal gene expression.^85^

O‐linked‐β‐N‐acetylglucosamine (O‐GlcNAc) transferase (OGT) is an enzyme that catalyses O‐GlcNAc to various cellular proteins. Cardiomyocyte‐specific deletion of OGT is characterized by cardiac hypertrophy in adult mice, suggesting that decreasing O‐GlcNAcylation induces hypertrophy development.^86^ However, emerging studies show that an increase in O‐GlcNAc levels was observed in pathological cardiac hypertrophy in the mice hearts induced by phenylephrine treatment.^87^, ^88^ Therefore, according to current reports, we cannot get a definite conclusion whether O‐GlcNAcylation induces or attenuates hypertrophy development and heart failure. Taken together, these findings further support our conclusion that O‐GlcNAcylation plays an important role in cardiac function and may be a therapeutic target.

## 7 ACETYLATION AND METHYLATION

**Figure 3:**
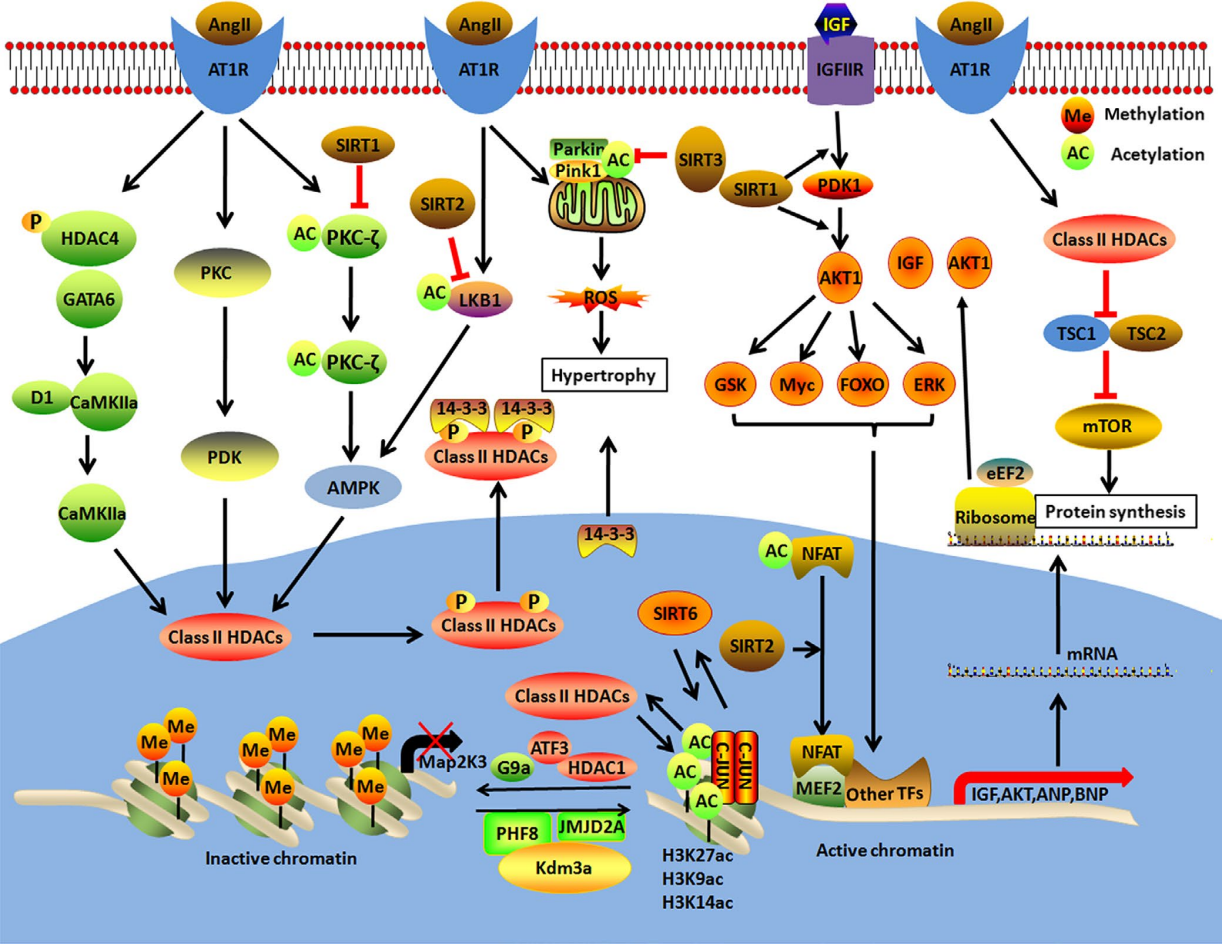
Acetylation‐ and methylation‐mediated signalling pathways of cardiac hypertrophy. Chromatin modifications are essential for regulating gene expression. Gene transcription can be regulated by acetylation and methylation of chromatin histones. Through remodelling the structure of chromatin, epigenetic modifications mediate the accessibility of DNA to regulate gene expression

Emerging evidence suggests that epigenetic modifications of histones, such as acetylation and methylation, are essential for the regulation of gene expression during the progression of cardiac hypertrophy.^89^ The correct expression of genes in cardiomyocyte is the basis for normal cardiac function. Thus, abnormal gene expression may cause heart dysfunction. Papait et al found that histone methyltransferase G9a regulated key epigenetic changes during the progression of cardiac hypertrophy (Figure 3). Hence, methylation was essential for cardiomyocyte homoeostasis and hypertrophy.^90^ Likewise, the histone trimethyllysine demethylase, JMJD2A, promoted cardiac hypertrophy in response to hypertrophic stimulation in mice and induced an increase in the expression of hypertrophy markers including B‐type natriuretic peptide and natriuretic peptide A in pluripotent stem cell‐derived cardiomyocyte (Table 5).^91^, ^92^ The histone demethylase, PHF8, was also observed to attenuate cardiac hypertrophy upon cardiac overload 93 (Figure 3).

**Table 5:** Roles of acetylation and methylation in myocardial hypertrophy

Name	Target and site	Result	Mechanism	Role in heart	Reference
G9a	H3K27 and H3K9	Inactivated chromatin	Repressed antihypertrophic genes via H3K9me2 and H3K27me3 deposition	Contributed to pathological cardiac hypertrophy	90
JMJD2A	H3K9me3	Activated chromatin	Activated transcription of cardiac foetal genes	Promoted pathological cardiac hypertrophy	91, 92
PHF8	H3K9me And H4K20me	Activated chromatin	Inhibited Akt‐mTOR pathway	repressed cardiac hypertrophy	93
HDAC4	Histone	Activated chromatin	Demethylated H3K9, dissociated HP1 and activated ANP gene	Promoted cardiomyocyte hypertrophy	95
HDAC1/2	Histone	Reduced TSC2 abundance	Activated mTOR signalling	Promoted pathological cardiac hypertrophy	98, 99
SITR1	PKC‐ζ	Inactivated PKC‐ζ	Suppressed activity of NF‐kB, ERK1/2 and ERK5	Prevented phenylephrine‐induced hypertrophic response	97
SITR2	LKB1	Activated LKB1	Activated AMPK signalling pathway	Inhibited ageing‐ and stress‐induced cardiac hypertrophy	108
SIRT3	Pink1/Parkin	Activated Pink/Parkin pathway	Induced mitophagy	Repressed cardiac hypertrophy	110
SIRT6	H3K9	Inactivated chromatin	Suppressed activity of c‐Jun	Attenuated cardiac hypertrophy	111

In the following section, we focus on the roles of acetylation in the development of cardiac hypertrophy progression (Figure 3). Previous studies reported the key function of histone deacetylases (HDACs) in the regulation of pathological heart growth. Class II HDACs maintain normal cardiac function and size by mediating the expression of MEF2 transcription factors and other factors.^94^ Recent studies reported that Class II HDACs were essential for vascular smooth muscle cell hypertrophy and hyperplasia through the CaMKIIα/protein kinase D1/HDAC4/GATA6 pathway.^56^ In addition, cardiomyocyte hypertrophy was attenuated by transcription factor 3 (ATF3), binding with the Map2K3 promoter, resulting in recruiting HDAC1 and suppressing MAP2K3‐p38 Signalling.^95^ Furthermore, the class III HDAC, sirtuin 1 (SITR1), reportedly prevented cardiomyocyte hypertrophy by negatively regulating the acetylation and phosphorylation levels of protein kinase C‐ζ (Table 5).^96^ Likewise, Class I HDACs attenuated cardiac hypertrophy by repressing the TSC2‐dependent mammalian target of rapamycin pathway.^97^, ^98^ Besides that, histone 3 at Lys9 (H3K9) was hyperacetylated upon ethanol exposure, inducing cardiac hypertrophy and ethanol‐induced cardiac hypertrophy was attenuated by an acardic acid in mice.^99^, ^100^ As the most abundant cells in mammalian heart tissue, cardiac fibroblasts contribute to cardiac remodelling and heart failure.^101^ In recent reports, HDAC inhibitors, in particular Class I HDAC inhibitors, were shown to attenuate pathological cardiac fibroblasts and ameliorate systolic and diastolic heart function in animal models.^102^, ^103^ For example, MGCD0103, a Class I HDAC inhibitor, was shown to inhibit cardiac fibrosis induced with angiotensin II via repression of ERK1/2 signalling.^104^ HDAC inhibitors were also shown to attenuate pathological cardiac hypertrophy.^98^, ^105^ These reports suggest that HDAC inhibitors may be the promising therapeutic drugs to treat heart failure. Although several reports show that pre‐clinical HDAC inhibitors are efficient in animal models of heart failure, no clinical trials using HDAC inhibitors are ongoing in heart failure patients. Four HDAC inhibitors (vorinostat, romidepsin, belinostat and panobinostat) have been approved by the FDA to treat cancer.^98^ In a recent systematic review, cancer patients treated with pan‐HDAC inhibitors exhibited mild cardiac side effects.^106^ Therefore, future work in this field is needed to delineate global cardiovascular safety of treatment with HDAC inhibitors in cancer patients.

SIRT2 was reported to act as a cardioprotective deacetylase by deacetylating liver kinaseB1 (LKB1) in pathological cardiac hypertrophy, resulting in activating AMPK signalling pathway.^107^ In addition, SIRT2 attenuated agonist‐induced cardiac hypertrophy by deacetylating NFATc2 transcription factor, leading to transcriptional suppression of hypertrophic genes.^108^ Recently, hypertension‐induced cardiac hypertrophy was reported to be protected by sirtuin 3 (SIRT3), deacetylating Pink1/Parkin, resulting in mitophagy and reduction of ROS production.^109^ Notably, sirtuin 6 (SIRT6) regulated the progression of cardiac hypertrophy by deacetylating H3K9 to inhibit IGF‐Akt signalling pathway (Table 5).^110^ SIRT6 also reported to prevent cardiomyocyte hypertrophy by inhibiting the expression of transcription 3 (STAT3).^111^ Finally, in SIRT6‐deficient hearts, SIRT1 was observed to be deacetylated and activated Akt signalling pathways.^112^

In conclusion, these findings highlight the critical role of both methylation and acetylation in the initiation, progression and outcome of maladaptive cardiac remodelling and dysfunction and HDAC inhibitors are promising drugs to target cardiac hypertrophic signalling for heart failure treatment.

## 8 THE MULTIFACETED CONTROL OF PTM

It is well recognized that cardiac hypertrophy is mediated at several levels, including gene transcription, processing and translation of mRNAs and PTMs. PTMs act as key regulators of proteins, occurring as a modification at a single residue or combining effects over multiple sites undergoing the same or different modifications.^113^ Cells need to be connected to various PTM signals and coordinated with each other to properly regulate cardiac hypertrophy. Furthermore, emerging evidence has highlighted important roles for crosstalk between different pairs of PTMs, such as ubiquitylation‐phosphorylation,^21^ SUMOylation‐phosphorylation,^65^ acetylation‐phosphorylation,^114^ O‐linked glycosylation‐phosphorylation,^80^ and acetylation‐methylation.^63^ For example, TAK1, an important signal transmitter, transmits the upstream signal from the receptor complex to the downstream signalling molecules. Recently, phosphorylation of TAK1 activated by NF‐κB was reported to contribute to further K63‐linked polyubiquitination modulated by TRIM8 in pathological hypertrophy 21 (Figure 1). SUMO‐1 is involved in heart failure by specifically mediating SUMOylation of SERCA2a. However, phosphorylation of SERCA2a is essential for SUMOylation of SERCA2a mediated by SUMO‐1 in mice and human patients with heart failure (Figure 2).^65^ AMPK is a hetero‐trimeric complex, which is activated by phosphorylation on the residue Thr172.^115^ In addition, AMPK inhibits O‐GlcNAcylation by mainly regulating phosphorylation of GFAT and AMPK activation counteracts cardiac hypertrophy by reducing O‐GlcNAcylation of proteins such as troponin T.^80^ Acetylation and trimethylation on H3K27 play opposing roles at the promoter regions of genes involved in cardiac hypertrophy.^116^ A previous study has shown that SIRT1 attenuated the PKC‐ζ activity via mediating the interplay of acetylation and phosphorylation in cardiac hypertrophy 96 (Figure 3). In conclusion, these findings suggest that crosstalk between different pairs of PTMs is essential for cardiac function. Future work in this field is needed to determine the global mechanistic actions of these PTMs in the heart.

## 9 CONCLUSIONS AND PERSPECTIVES

A considerable number of studies have shown that myocardial hypertrophy is a phenomenon in which cardiac cells transform from a mature ‘contractile state’ to an ‘embryonic synthesis state’ and is the primary pathophysiological process in the development of heart failure.^117^ Myocardial hypertrophy can lead to reduced blood pressure, cardiac cell hypertrophy and apoptosis, decreased ventricular compliance and impaired ejection function, resulting in a vicious cycle of worsening cardiac functions. Overall, myocardial hypertrophy has become an increasingly important factor in the field of cardiovascular disease. Therefore, it is particularly important to explore its mechanism.

As reported in the studies reviewed in this article, myocardial hypertrophy is connected with various cellular signalling pathways and PTMs. PTMs of proteins can precisely regulate and improve the stability and activity of diverse signalling pathways. PTMs are closely related to the occurrence and developmental process of cardiac hypertrophy, but their molecular mechanisms and regulatory network still remain elusive and require further investigations. Therefore, a thorough investigation of the regulatory mechanisms of PTMs in the process of cardiac hypertrophy can help us better understand the basis of myocardial hypertrophy and develop improved drugs to prevent or reverse this disorder.

## CONFLICTS OF INTEREST

The authors declare no conflict of interest.
